# Physiological expression of olfactory discrimination rule learning balances whole‐population modulation and circuit stability in the piriform cortex network

**DOI:** 10.14814/phy2.12830

**Published:** 2016-07-22

**Authors:** Luna Jammal, Ben Whalley, Sourav Ghosh, Raphael Lamrecht, Edi Barkai

**Affiliations:** ^1^ Sagol department of NeurobiologyFaculty of Natural SciencesUniversity of HaifaHaifaIsrael; ^2^ School of Chemistry, Food & Nutritional Sciences and PharmacyThe University of ReadingReadingUK

**Keywords:** Brain slices, lomg‐term memory, piriofm cortex, brain slices, synaptic plasticity

## Abstract

Once trained, rats are able to execute particularly difficult olfactory discrimination tasks with exceptional accuracy. Such skill acquisition, termed “rule learning”, is accompanied by a series of long‐lasting modifications to three cellular properties which modulate pyramidal neuron activity in piriform cortex; intrinsic excitability, synaptic excitation, and synaptic inhibition. Here, we explore how these changes, which are seemingly contradictory at the single‐cell level in terms of their effect on neuronal excitation, are manifested within the piriform cortical neuronal network to store the memory of the rule, while maintaining network stability. To this end, we monitored network activity via multisite extracellular recordings of field postsynaptic potentials (fPSPS) and with single‐cell recordings of miniature inhibitory and excitatory synaptic events in piriform cortex slices. We show that although 5 days after rule learning the cortical network maintains its basic activity patterns, synaptic connectivity is strengthened specifically between spatially proximal cells. Moreover, while the enhancement of inhibitory and excitatory synaptic connectivity is nearly identical, strengthening of synaptic inhibition is equally distributed between neurons while synaptic excitation is particularly strengthened within a specific subgroup of cells. We suggest that memory for the acquired rule is stored mainly by strengthening excitatory synaptic connection between close pyramidal neurons and runaway synaptic activity arising from this change is prevented by a nonspecific enhancement of synaptic inhibition.

## Introduction

Rats trained to perform a particularly difficult olfactory discrimination (OD) task demonstrate a dramatic increase in their capability to acquire memories of new odors, once they have learnt the first discrimination task (“rule learning”) (Saar et al. 1998, 1999). Such rule learning is accompanied by a series of long‐lasting cellular modifications in layer II pyramidal neurons of the piriform cortex. Long‐term enhancement occurs in three components controlling neuronal activation; excitatory synaptic drive mediated by glutamate receptors (Saar et al. 1999, 2002, 2012; Knafo et al. 2005; Ghosh et al. 2016), intrinsic neuronal excitability (Saar et al. 1998, 2001; Cohen‐Matsliah et al. 2007), and GABA receptor‐mediated synaptic inhibition (Brosh and Barkai 2009; Saar et al. 2012; Kfir et al. 2014; Ghosh et al. 2015). In particular, enhanced excitatory and inhibitory synaptic transmission is strongly expressed on the fourth and fifth day after rule learning (Saar et al. 2012; Ghosh et al. 2015, 2016).

Enhancement of synaptic inhibition is required to compensate for the concurrently enhanced excitability which could lead to epileptiform discharges (Hasselmo and Barkai 1995; Golomb and Ermentrout 2002) to which the piriform cortex is prone (Vismer et al. 2015). Also, the predisposition for long‐term potentiation (LTP) induction is reduced and that for long‐term depression induction in intrinsic fibers interconnecting layer II pyramidal neurons is enhanced 1 day after learning (Lebel et al. 2001), in accordance with the sliding modification threshold theory (Bear 1996). This effect is mediated by a transient modification in NMDA‐channel subunit composition, resulting in receptors that have a higher complement of the NR2A subunit protein relative to NR2B (Quinlan et al. 2004).

While the cellular and subcellular processes that subserve learning‐induced modifications continue to be characterized, the relationship between such single‐cell changes and modifications to network function which ultimately dictate piriform cortex output have not yet been studied. In particular, it is unclear how modifications that exert opposing effects at the single‐cell level (enhanced intrinsic excitability and enhanced excitatory synaptic transmission vs. enhanced inhibitory synaptic transmission and reduced susceptibility for LTP induction) manifest themselves within the piriform cortical network to maintain rule memory.

The piriform cortex is well suited for multiple fPSPs recordings from well‐identified cellular locations. The piriform cortex receives direct input from the olfactory bulb, via the lateral olfactory tract, without thalamic intervention. It has a simple and well‐defined anatomical organization: pyramidal cell bodies are densely packed in a thin layer (layer II), with intracortical association axons synapsing at the proximal zone of apical dendrites (layer Ib), and afferent input axons from the olfactory bulb synapsing at the distal part of apical dendrites (layer Ia). This laminar organization is easily visualized and enables simultaneous recordings from homogenous populations of neurons and specific activation of well‐defined synaptic pathways when acute slices are cut in transverse or coronal planes.

The inputs to the piriform cortex from the olfactory bulb are nontopographically spread across the entire surface of the piriform cortex. Accordingly, presentation of eight different odors to rats results in an increased firing rate ~30% of piriform cortex cells, with each cell responding to at least one odor (Schoenbaum and Eichenbaum 1995). This unique, widespread activation results from overlapping afferent inputs and hardwired connectivity between pyramidal neurons (Johnson et al. 2000) where widespread axonal arbors of individual neurons extend over nearly the full length of the cerebral hemisphere, unlike the columnar organization of other primary sensory areas which are associated with regularly arranged, patchy concentrations of connectivity (Haberly 2001; Franks et al. 2011). Thus, each pyramidal neuron makes a small number of synaptic contacts with a large number of other cells at disparate locations in the piriform cortex.

Here, we combine multisite recording of simultaneous fPSPs and whole‐cell patch‐clamp recordings to demonstrate the manner by which learning‐induced modifications in single neurons modify the neuronal ensemble activity.

## Materials and Methods

### Animal training

#### Subjects and apparatus

**Figure 1:**
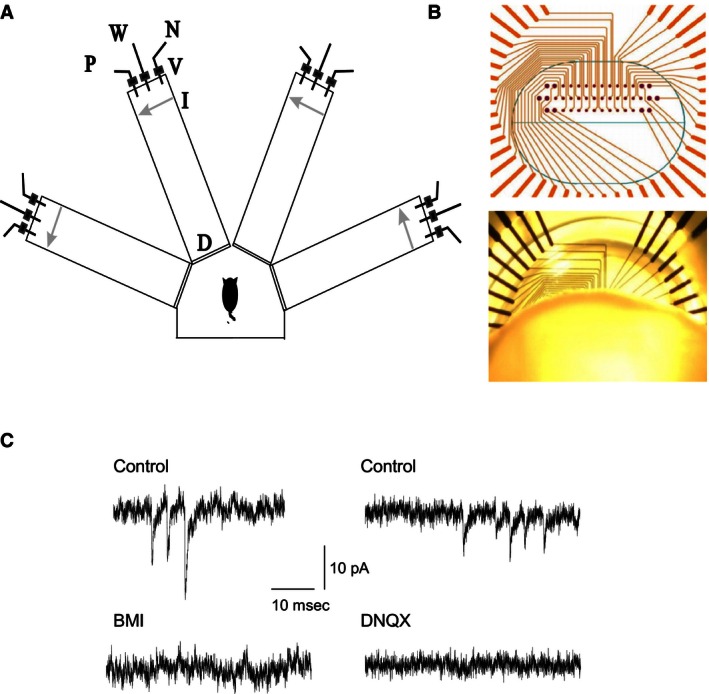
Training and elecrophysiological recordings. (A) Schematic representation of the olfactory maze. Protocols for “trained” and “pseudotrained” rats were similar: an electronic “start” command randomly opened two of eight valves (V), releasing pressured air streams with positive cue odor (P) into one of the arms and negative‐cue odor (N) into another. Eight seconds later, the two corresponding guillotine doors (D) were lifted to allow the rat to enter the selected arms. Upon reaching the far end of an arm (90 cm long), the rat's body interrupted an infrared beam (I, arrow) and a drop of drinking water was released from a water hose (W) into a small drinking well. For a “trained” rat, this occurred only if the arm contained the positive cue odor; for a “pseudotrained” rat, it occurred randomly. A trial ended when the rat interrupted a beam, or after 10 sec if no beam was interrupted. A fan operated for 15 sec between trials, to remove odors. Each rat underwent 20 trials per day. (B) Placement of piriform cortex slices on microelectrodes arrays (MEAs). Top: Thirty‐two recording electrodes (small dots) are arranged in three rows, with distance of 90 mm between electrodes on the same row and 150 mm between electrodes on the same column. Twelve stimulating electrodes (large dots) surround the recording electrodes. Bottom: a coronal piriform cortex slice mounted on a MEA. Schematic layer topography of the piriform cortex is shown. Slices were positioned on the MEA such that cell bodies layers (Layer II) were aligned with the bottom row of MEA recording electrodes. This positioning allowed recording of signals from layer Ib proximal dendrites via the second row of electrodes and signals from more distal dendrites via the first row of electrodes. (C) Recordings of spontaneous miniature postsynaptic currents. Left: Miniature inhibitory synaptic events recorded from a neuron at a holding potential of −60 mV. Excitatory synaptic activity was abolished by the specific GABA_A_ receptor blocker, bicuculline. Right: Miniature excitatory synaptic events recorded from a neuron at a holding potential of ‐80 mV. Inhibitory synaptic activity was abolished by the specific AMPA receptor blocker, DNQX.

Age‐matched young adult (~150 grams at the beginning of training), Sprague–Dawley male rats were used. Prior to training, rats were maintained on a 23.5 h water‐deprivation schedule, with food available ad libitum. The olfactory discrimination training protocol was performed daily on each trained and pseudotrained rat in a 4‐arm radial maze (Fig. 1A) as previously described (Saar et al. 1999, 2001) using commercial odors (Lemon, Peach, Orange and Pineapple) that are regularly used in the cosmetics and food industry.

#### Training

Olfactory training consisted of 20 trials per day for each rat as previously described (Saar et al. 2001). In short, in each trial, the rat had to choose between two odors (positive and negative cue) presented simultaneously. Rats designated to the trained group were rewarded with drinking water upon choosing the positive cue. Rats in the pseudotrained group were rewarded in a random fashion, upon choosing any odor. The criterion for learning was at least 80% positive cue choices in the last 10 trials of a training day (Saar et al. 1999, 2001). Rats in the naïve group were water deprived, but not exposed to the maze.

Our previous studies have shown that the two learning phases can be clearly distinguished (Saar et al. 1998, 1999; Zelcer et al. 2006): during the first phase of rule learning, which usually requires 7–8 days, rats develop a strategy for performing the odor discrimination task while during second phase of enhanced learning capability, rats can learn new odors within 1–2 training days. In this study, rats were trained with one pair of odors, until they reached the criterion for rule learning. They were subsequently allowed to rest for 4–5 days, after which the brain slices were prepared.

### Slice preparation and recordings

#### Slice preparation

Rats were anesthetized using pentobarbital (30 mg/kg). Brain slices were taken from the posterior piriform cortex. A length of 400 *μ*m coronal brain slices for multisite extracellular recordings and 300 *μ*m slices for whole‐cell patch clamp recordings were cut as previously described (Saar et al. 1998, 2012) and kept for 1 h in oxygenated (95% O_2_ + 5% CO_2_) normal saline Ringers’ (N.S.R.) solution (in mmol/L: NaCl 124, KCl 3, MgSO42, NaH2PO4 1.25, NaHCO3 26, CaCl_2_ 2 and glucose 10). For single‐cell recordings, slices were placed in a recording chamber under an infrared DIC microscope, and perfused with Ringer's solution at 30°C. Whole‐cell voltage‐clamp recordings were obtained from visually identified pyramidal neurons in layer II of the piriform cortex. All electrophysiological recordings were performed using Axopatch 1D (Molecular Devices, Sunnyvale, CA), and data were acquired using pClamp9 (Molecular Devices).

The experimenter was blinded to the identity of the rat (naive, trained, or pseudotrained) from which neurons were recorded.

#### Microelectrodes array (MEA) recordings

A total of 22 trained rats, 18 naïve rats and 19 pseudotrained rats were used for fPSPs recordings with MEA.

Electrical activity of each piriform cortex slice was monitored and recorded using perforated MEAs (Fig. 1B). Each MEA (Multi Channel Systems GmbH, Reutlingen, Germany) comprised 32 planar recording electrodes each of 30 *μ*m diameter, arranged in three layers with and interelectrode distances of 90 *μ*m (within a layer) and 150 *μ*m (between layers). Prior to recording, MEAs were cleaned with ethanol and then rinsed with distilled water. Slices were attached to the perforated MEA using negative pressure applied with a vacuum system. Slice position on the MEA was ascertained by observation using a binocular microscope (Motic, Hong Kong, China) at magnification ×4 and images of the slice and electrode positions acquired via a camera (Moticam 2000, 2.0M pixel USB2.0) to a PC. Once attached, slices were continually perfused with carboxygenateda CSF (2 mL/min) and maintained at 30°C in order to provide good separation of synaptically mediated LFP components (Saar et al. 1999). fPSPs were evoked via one of twelve stimulating electrodes (Fig. 1B). Signals were amplified (1200 ×  gain) by a 120‐channel dual‐head stage amplifier (USB‐MEA‐32‐STIM4 amplifier; Multi Channel Systems GmbH, Reutlingen, Germany) and each channel simultaneously sampled at a minimum of 10 kHz. Data was acquired on a PC using LTP‐Analyzer software (Multi Channel Systems GmbH) to monitor and record data for subsequent offline analysis.

#### LTP induction and measurement

LTP was induced by applying six cycles of theta bursts (5 Hz) to the intrinsic fibers. Each cycle consisted of 10 such bursts, each entailing four stimuli at 50 Hz. Thus, a total of 240 stimuli were applied for LTP induction. The extent of potentiation was defined as the ratio between the amplitude of the fPSP evoked every 10 sec before and after the tetanic stimulation. LTP values were calculated by averaging the traces recorded at the 20 min after application of the tetanus stimuli.

#### Spontaneous synaptic events

##### Miniature inhibitory postsynaptic currents (mIPSCs) recordings

To record GABA_A_‐mediated mIPSCs, the recording electrode contained (in mmol/L): 140 CsCl, 1 EGTA, 6 KCl, 4 NaCl, 2 MgCl_2_, and 10 HEPES.pH = 7.25, 280 mOsm. Under these conditions, the reversal potential of chloride was ~0 mV and thus strong GABA_A_‐mediated currents could be studied at holding potential of −60 mV (Fig. 1C). The perfusion solution contained TTX (1 *μ*mol/L) plus DNQX (20 *μ*mol/L) as previously described in addition to APV (50 *μ*mol/L), to block glutamatergic synaptic transmission via AMPA receptors in order to allow recording of isolated mIPSCs. Isolation of mIPSCs was confirmed by abolition with BMI (20 *μ*mol/L; Fig. 1C).

During continuous recordings, the current response to 200 ms voltage steps of −5 mV applied at 1 Hz was monitored. A change in the response caused exclusion of the data.

For data analysis, each event was detected by eye, and measured using “Mini Analysis” software (SynaptosoftInc., Decatur, GA).

##### Miniature excitatory postsynaptic currents (mEPSCs) recordings

Cells were voltage clamped at Vm = −80 mV, which has previously been determined as the normal resting potential for this population based on intracellular microelectrode current clamp recordings (Saar et al. 1998). At this membrane potential, most voltage‐dependent channels are closed and NMDA receptors are rarely activated.

The recording electrode was filled with a solution containing (in mmol/L): 140 K‐gluconate, 1 EGTA, 6 KCl, 4 NaCl, 2 MgCl_2_, and 10 HEPES. pH = 7.25, 280 mOsm. mEPSCs were recorded in the presence of 1*μ*mol/L TTX (Fig. 1C). At the end of each experiment, additional recording (10 min) was performed with 20 *μ*mol/L DNQX in the perfusing solution, to ensure that there was no contamination of the data acquired with non‐AMPAR‐mediated events. Given the low concentration of chloride in the patch pipette solution (10 mmol/L), the reversal potential of GABA‐mediated IPSCs would be around −65 mV, and thus miniature IPSCs at Vm = −80 mV would be very small. Indeed, after DNQX application, no spontaneous events were observed in any of the recordings, indicating that only AMPAR‐mediated currents were measured (Fig. 1C).

### Statistical analysis

Between‐groups comparisons were done using a one‐way analysis of variance (ANOVA) test, and posthoc multiple nondirectional *t*‐tests were then applied to compare between groups. Values throughout the text are presented as mean ± SD. Data in graphs are presented as mean ± SE. Comparison of cumulative frequency curves describing IPSCs and EPSCs amplitudes distributions (Fig. 8) were conducted using the Kolmogorov–Smirnov test.

## Results

### Spatial distribution of evoked synaptic responses is not modified by learning

**Figure 2:**
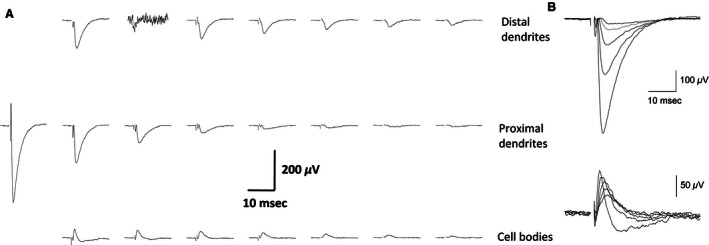
Examples of simultaneous fPSPs recordings from a piriform cortex slice (A) Stimulus was applied 100 μm left to the first response on the second row (layer Ib). Note the rapid decline in fPSP amplitude as the distance from the point of stimulation increases. Also, note that while the responses in the two upper rows, which represent recordings from apical dendrites are negative going, indicating inward synaptic currents, responses in the cell body layer are positive, indicating outward currents. Recording are shown in 22 out of 32 recording electrodes, where fPSPs are detected. Slice was taken from a pseudotrained rat. (B) Responses to a set of increasing stimulus intensity recorded from the second electrode in the second row and the first electrode from the third row (the first column from the left). The amplitude of both fPSP increases with the increase in stimulus intensities, but inward synaptic currents are maintained restricted to the dendrites.

**Figure 3:**
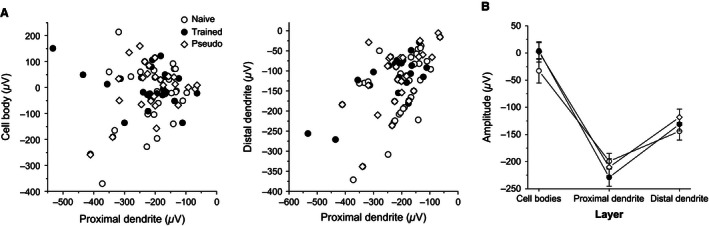
Responses in the cell bodies layer are isolated from responses in the dendrites (A) For the three groups, no correlation was found between the amplitude of responses in the proximal dendrites and the cell bodies (left figure, for naïve *R* = 0.22, *P* = 0.28, for trained *R* = 0.05, *P* = 0.79, for pseudotrained *R* = 0.28, *P* = 0.15). A strong correlation was found between amplitude of responses in the proximal and distal dendrites (for naïve *R* = 0.48, *P* = 0.01, for trained *R* = 0.62, *P* < 0.001, for pseudotrained *R* = 0.56, *P* = 0.002). (B) A summary of depth profiles for the three groups. fPSPs used here are taken from the closest column to the stimulating electrode (closest recordings from the first and third layers, second recording from the second layer). Recordings were made using a stimulus intensity of 50% of that required to elicit a maximal fPSP in the second layer. The depth profile is similar for the three groups. The averaged values of near 0 for the cell bodies layer results from variations in the responses in different slices, which were in some slices positive and negative in others. Data were recorded from 27 slices taken from 22 naïve, 34 slices taken from 26 trained, and 27 slices taken from 22 pseudotrained rats. Values represent average ± SE.

**Figure 4:**
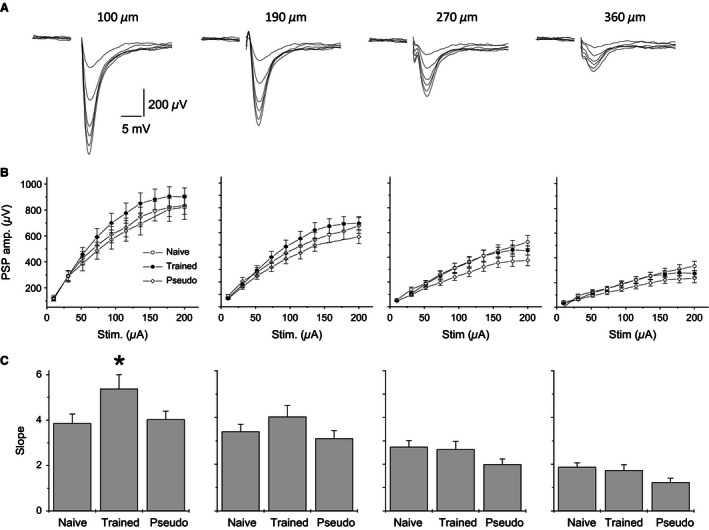
Learning‐induced enhancement of synaptic connectivity is apparent between proximate neurons. (A) fPSPs recorded simultaneously via four electrodes, located at the proximal dendrites, at increasing distances from the stimulation site and in response to progressively increasing stimulus intensities. Stimuli were applied via an electrode in piriform cortex layer *I*_b_. Distances between recording electrodes and the stimulus location are indicated above each recording. Increased stimulus intensity results in a nonlinear increase in fPSPs amplitudes. (B) Input/output (I/O) curves obtained from recordings of the type described in A. Values represent average ± SE. (C) The linear component of the I/O curve slope is significantly steeper (*P *<* *0.05) in slices from trained rats at recording sites most proximal to the stimulating electrode site. Values represent average ± SE. *P < 0.05.

When applying focal stimulation to association fibers via MEA electrodes with a stimulus intensity that elicited a response of 50% from the maximal stimulation intensity used for generating the input/output curve (I/O curve, see Fig. 4), a clear spatial distribution of responses was always observed along both the vertical axis, recording activity between different layers and the horizontal axis, recording activity at different distances along the same layer. Figure 2 shows the evoked local field potentials recorded simultaneously throughout the array in response to application of a stimulus to layer Ib. The short delay, 5–7 msec, to the first field synaptic response (fPSP) indicated that a monosynaptic response was being evoked (Saar et al. 1999). Potentials recorded from the two rows of electrodes that were parallel to the distal and proximal dendritic trees always revealed negative going responses indicating a net transfer of positive charge away from the recording electrode and consistent with an inward, excitatory synaptic current. Recordings made from the row of electrodes situated parallel to layer II pyramidal cell bodies while stimulating in layer Ib differed by revealing only a positive going response (Fig. 2A), indicating that the evoked synaptic response is restricted to the dendritic inputs. In some other recordings, a negative fPSP was observed also in the cell bodies layer. In addition to the layer‐specific differences in responses to electrical stimulation already noted, the amplitudes of evoked synaptic responses decreased with increasing distance from the stimulating electrode in each of the three rows of electrodes. Moreover, while increasing stimulus intensity resulted in increased response amplitudes at each of the three recording depths, the direction (positive/negative) of each response was unaltered at each depth (Fig. 2B), indicating that the net inward synaptic currents remain restricted to the dendrites. Further indication that the synaptic responses decay passively to the cell body layer was found in the strong correlation between the response amplitudes of the two dendritic recording locations, but no such correlation between response amplitudes of the dendritic and cell body responses in recordings from all three experimental groups (naïve, trained, and pseudotrained; Fig. 3A). Moreover, the average responses in a given layer did not differ between groups (Fig. 3B).

### Learning‐induced modulation of synaptic responses

To explore the possible modifications in synaptic excitability arising from training, input‐output (I/O) curves were constructed for fPSPs recorded simultaneously from four locations (Fig. 4A and B). The slopes of the I/O curves serve as an indication of the synaptic excitability in the examined pathway (Saar et al. 1999; Cohen et al. 2008). We have previously shown that 3 days after rule learning, enhanced synaptic excitability can be detected in piriform cortex brain slices (Saar et al. 1999, 2002). Here, in the first electrode located 100 *μ*m from the stimulating electrode, the average slope of the linear portion of the I/O curve in slices from trained rats (5.38 ± 2.7 V/A, *n* = 19 slices from 17 rats) was significantly (*F*_(2,45)_ _=_ 3.2, *P* < 0.05) higher than the average in slices from naïve (3.86 ± 1.8, *n* = 20 slices from 16 rats) and pseudotrained rats (4.02 ± 1.5, *n* = 18 slices from 15 rats), neither of which differed from one another (Fig. 4C). However, the average slopes of the I/O curves in the second (trained: 4.03 ± 2.1; naïve: 3.40 ± 1.4; pseudotrained: 3.1 ± 1.5V/A), third (trained: 2.63 ± 1.5; naïve: 2.74 ± 1.4; pseudotrained: 2.63 ± 1.5V/A), and fourth (trained: 1.73 ± 1.0; naïve: 1.87 ± 0.8; pseudotrained: 1.2 ± 0.8 V/A) electrodes, which were located at distances of 190, 270, and 360 *μ*m from the stimulating electrode respectively, did not differ between groups. Therefore, we show that 5 days after learning, synaptic enhancement is apparent, but only in synapses located close to the stimulating electrode.

### High‐frequency stimulation results with uniform LTP throughout the cortical network

We next examined how the susceptibility for LTP induction may be modified with increasing the distance between stimulating and recording electrodes. LTP was induced by repetitive 50 Hz stimulation. Our previous studies have shown that one day after learning, LTP induced by such stimulation is significantly lower in slices from trained rats (Lebel et al. 2001; Quinlan et al. 2004).

**Figure 5:**
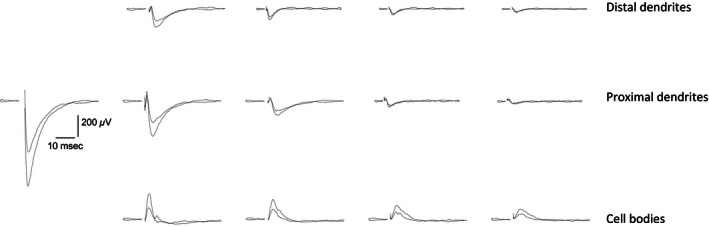
LTP induction in the piriform cortex slice. LTP was induced by repetitive 50 Hz stimulation. Example shown was recorded from a slice taken from a naïve rat. Superimposed traces were taken just prior to repetitive stimulation and at 20 min afterwards. fPSP were recorded in response to low‐frequency stimulation at 0.1 Hz. LTP was apparent in the three recorded layers at distance of several columns. Poststimulus responses are larger in all recordings locations. Notably, at sites where LTP was observed, fPSPs maintained their initial polarity. Locations of each electrode row are indicated.

**Figure 6:**
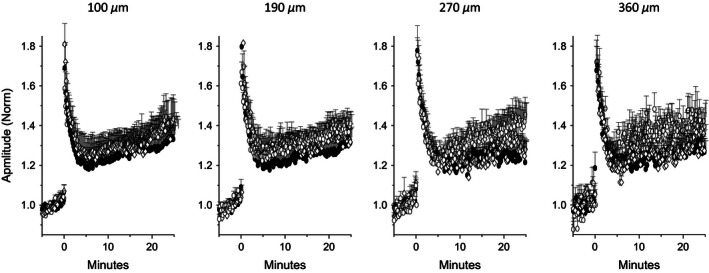
MEA recordings of LTP in the piriform cortex slices Amplitudes of fPSPs recorded at four locations simultaneously, normalized to the evoked fPSP amplitude prior to administration of high‐frequency stimulation. Although the reproducibility of responses decreased with increasing distance from the stimulating electrode as a result of the decline in the initial amplitude (see Fig. 2), LTP can still be induced to the same extent at all four locations Open circles: naïve, filled circles: trained, open diamonds: pseudotrained. Values represent average ± SE. Data were recorded in 16 slices taken from 15 naïve, 14 slices taken from 8 trained and 11 slices taken from 9 pseudotrained rats..

**Figure 7:**
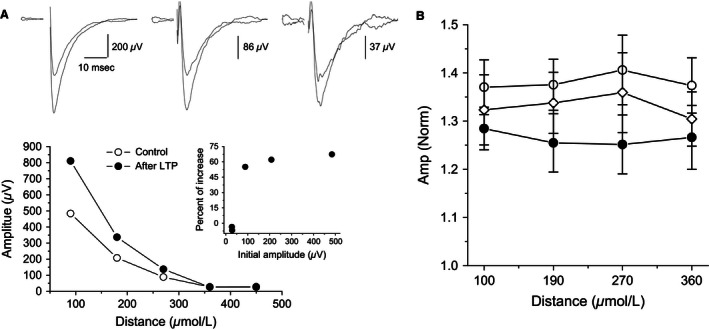
LTP amplitude is uniform throughout the cortical slice (A) Top: Recordings from three layer II adjacent electrodes before and after LTP induction, with amplitudes normalized to the fPSP closest to the stimulation before LTP induction. Bottom Values of measured fPSPs recorded from five layer II recording electrodes, before and after LTP induction. Inset: average LTP recorded from the five electrodes; LTP was similar in all location where it could be induced. Traces were recorded in a slice taken from a naïve rat. (B) Quantitative description of LTP as a function of distance from the stimulating electrode. For three groups, LTP was induced to the same value, regardless of the distance from the stimulating electrode. While LTP had a somewhat lesser averaged value in slices form trained rats, the differences between the three groups did not reach the level of significant difference. Open circles: naïve, filled circles: trained, open diamonds: pseudotrained. Values represent mean ± SE. Graphs describe data from the same recordings shown in Figure 6.

High‐frequency repetitive stimulation results in LTP induction at susceptible synapses, usually throughout recording sites where the initial response was detected (Fig. 5). Moreover, although the initial amplitude of the responses declined steeply with the increasing distance between the stimulating and the recording electrodes, the extent of LTP was uniform for all synaptic responses (Figs. 6 and 7). The averaged LTP amplitude in fPSPS from trained rats (data for calculating the LTP amplitude was pulled from the four potentials recorded simultaneously in each slice) was 1.27 ± 0.23 (*n* = 60 slices from trained rats). Although this value was seemingly lower than the averaged LTP amplitude recorded in slices from control rats (naïve [*n* = 56]: 1.38 ± 0.21; pseudotrained [*n* = 41]: 1.34 ± 0.27), the difference between the three groups stopped short from reaching significant value; the one‐way ANOVA indicated a significant difference (*F*_(2,155)_ = 3.4, *P* < 0.05), but the post hoc test yielded *P* = 0.17 when amplitudes recorded in slices form trained and pseudotrained rats were compared.

Interestingly, the averaged LTP amplitude was similar in all recorded electrodes in any particular slice, and the averaged values of LTP were similar also in the four recording electrodes for each group (Figs. 6 and 7). As such, LTP uniformity was detected regardless of large differences between fPSP amplitudes recorded at different distances from the stimulating electrode (Fig. 7A).

### Learning enhances excitatory and inhibitory synaptic transmission to the same extent

The minor differences in evoked synaptic responses between slices from trained and control rats, as detected here 5 days after learning, is somewhat surprising in face of the prior knowledge that during this time, robust enhancement of excitatory and inhibitory synaptic transmission in the piriform cortex network is seen (Saar et al. 2012; Kfir et al. 2014; Ghosh et al. 2015). We therefore examined the possibility that these two forms of synaptic enhancement balance one another at the network level. To this end, we recorded miniature excitatory and inhibitory synaptic events from layer II pyramidal neurons and compared the magnitudes by which they were enhanced after learning.

**Figure 8:**
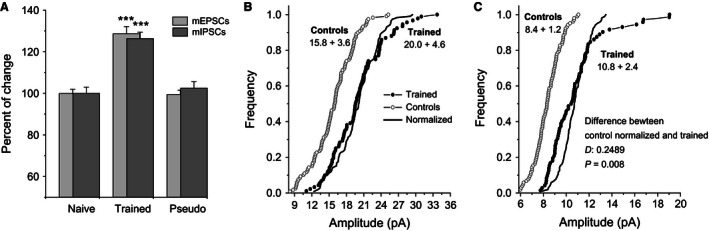
Learning‐induced enhancement of the inhibitory and excitatory synaptic currents (A) Averaged amplitude of spontaneous miniature inhibitory and excitatory synaptic events normalized to the averaged control values (amplitudes of events recorded from naïve plus pseudotrained rats). For both inhibitory and excitatory events, responses in neurons from trained rats are significantly higher (*P < 0.05) than in neurons from either control group. The averaged amplitude was calculated for each neuron from all spontaneous events. Values represent Mean + SE. One neuron was recorded in each slice and up to four neurons were recorded from slices from the same rat. (B) Cumulative frequency distributions of inhibitory event amplitudes in neurons from trained and controls. Each point represents the averaged event amplitude in a neuron. Note that the averaged IPSC amplitude appears to be equally increased in most neurons in the trained group. Indeed, an addition of 4.2 pA, the averaged difference between trained and control neurons, to all values in the control group resulted with a graph that precisely overlaps the cumulative graph describing the trained neurons. (C) Cumulative frequency distributions of excitatory event amplitudes in neurons from trained and controls. Each point represents the averaged event amplitude in a neuron. Here, the increase in the EPSC amplitude seems to be distributed nonequally between the neurons; it is particularly expressed in a subgroup of cells. Accordingly, an addition of 2.4 pA, the averaged difference between trained and control neurons, to all values in the control group resulted with a graph that significantly different from the cumulative graph describing the trained neurons.

As previously reported (Saar et al. 2012; Ghosh et al. 2015, 2016), olfactory discrimination learning induced dramatic increase in the amplitudes of both excitatory and inhibitory miniature synaptic events which were apparent throughout the neuronal cell population. This was confirmed in our recordings here where the average mIPSC amplitude in neurons from trained rats (20.0 ± 4.6 pA, *n* = 92 cells from 50 rats) was significantly (*F*_(2,204)_ = 27.67, *P* < 0.001) higher than in pseudotrained (16.0 ± 3.6pA, *n* = 58 cells from 40 rats) or naïve rats (15.6 ± 3.6 pA, *n* = 61 cells from 38 rats). Similarly, mEPSC amplitude in neurons from trained rats (10.8 ± 2.4 pA, *n* = 72 cells from 41 rats) was significantly (*F*_(2,174)_ = 38.05, *P* < 0.001) higher than in neurons from pseudotrained (8.33 ± 1.2pA, *n* = 49 cells from 35 rats) or naïve rats (8.37 ± 1.2pA, *n* = 55 cells from 40 rats). Notably, the most striking finding here was the similarity between the extents of the enhancement of the two types of synaptic transmission examined (nondirectional *t* test, *P* = 0.61); average mEPSC amplitude was increased by 28.7% after learning while average mIPSC amplitude was increased by 26.4% (Fig. 8A).

### Difference between synaptic inhibition and excitation in the distribution of learning‐induced strengthening

A closer examination of the distributions of the amplitudes of the spontaneous synaptic events, showed a significant difference between inhibition and excitation in the manner by which learning‐induced synaptic enhancement affects the pyramidal cells population.

A Kolmogorov–Smirnov test showed that the increase in the averaged mIPSC amplitude can be explained by a simple additive process; adding a value of 4.2 pA (26.4% of the average in controls) to each of the measured mIPSCs in neurons from control (naïve and pseudotrained) rats resulted with a cumulative frequency curve that did not differ (*P* = 0.16) from that describing the values measured in neurons from trained rats (Fig. 8B). In contrast, when such a simple addition process was applied to the mEPSCs values recorded in neurons from control rats (added value of 2.4 pA, 28.7% of the average in controls), a Kolmogorov–Smirnov test showed that the calculated curve differed significantly (*P* < 0.01) from the curve describing the values recorded in neurons from trained rats (Fig. 8C). In particular, it appears that a subgroup of excitatory synapses is particularly enhanced after learning.

## Discussion

Our previous studies show that OD rule learning is accompanied by a series of pre‐ and postsynaptic cellular modifications. These modifications have three major traits:

- They are widespread throughout the piriform cortex network; physiological and morphological modifications are found in most of the studied neurons (Saar et al. 1998, 2002, 2012; Saar and Barkai 2003; Knafo et al. 2005).
- The time course during which these modifications manifest and decline is strongly correlated with skill acquisition (Barkai 2005). However, memories for specific odors far outlast these modifications.
- In parallel to increased excitability, synaptic inhibition is also enhanced after learning (Brosh and Barkai 2009; Saar et al. 2012; Ghosh et al. 2015) and the subunit composition of NMDA receptors is temporarily modified in manner that favors activity‐induced synaptic weakening over enhancement (Lebel et al. 2001; Quinlan et al. 2004).

Here, we examined how the combination of these single‐cell modifications is expressed at the network level. Our data show that the cortical network largely maintains its stability in the face of the combined cellular changes, and the only detectable net change is in enhanced excitatory synaptic connectivity between spatially proximal neurons. Such stability may be explained at least partially by the finding that excitatory and inhibitory synaptic transmission is enhanced almost identically after learning.

### Evoked synaptic responses are much localized

While it is difficult to estimate the number of neurons that contribute to each recorded fPSP, the stimulating electrode in our MEA device allows localized stimulation and therefore the recorded responses arise from activity generated proximal to the recording electrode (Steidl et al. 2006; Hill et al. 2010). Indeed, our data show that while there was a clear correlation between the amplitudes of fPSPs recorded at proximal and distal dendrites, the amplitudes of fPSPs recorded at the cell bodies layer are not correlated with any fPSPs recorded at vertically proximal dendritic components. Since the vertical distance between MEA recording electrodes is 150 *μ*m, the lack of cell body‐dendrite fPSP correlation indicates that synaptic responses are predominantly localized. The finding that the strength of synaptic connectivity decays steeply with increasing distance between stimulation and recording sites is somewhat surprising in light of the piriform cortex anatomy. Piriform cortex individual pyramidal cells have widespread axonal arbors that extend over nearly the full length of the cerebral hemisphere, with no regularly arranged patchy concentrations like those associated with the columnar organization in other primary sensory areas (Haberly 2001; Franks et al. 2011).

### Stability of spatial distribution of localized synaptic responses

The similarity in the averaged depth profiles of the recorded responses between the three animal groups (Fig. 3B) indicates that the isolation between the cell body and the dendritic tree in layer II pyramidal neurons is not modified by learning. In addition, the vertical propagation of synaptic responses, that can be used to evaluate changes in network excitability (Golomb and Amitai 1997; Golomb and Ermentrout 2002; Kfir et al. 2014), is modified only moderately after learning. Synaptic responses decayed quickly with distance and this decay was not modified after learning. Moreover, I/O curves showed that local excitability remained stable with the sole exception off PSPs recorded from electrodes closest to the stimulating electrodes.

Notably, our recordings were performed 5 days after learning, when intrinsic neuronal excitability and dendritic spine number have resumed their pretraining values (Saar et al. 1998; Knafo et al. 2005). Thus, we suggest that long‐term net increase in synaptic excitation is most pronounced in connections between close neurons.

### Stability of predisposition for LTP induction

Our previous studies showed that OD rule learning produces a reduced predisposition for LTP induction (Lebel et al. 2001; Quinlan et al. 2004). This effect is mediated by modification in the subunit composition of the NMDA receptor, which persists for fewer than 3 days after rule learning (Quinlan et al. 2004).Thus, it is not surprising that 5 days after rule learning, LTP magnitude does not differ between slices from trained and control rats.

An unexpected finding is that the magnitude of LTP is similar in all locations where an fPSP was detected, regardless of the initial recorded amplitude. LTP in the piriform cortex is NMDA‐dependent (Kanter and Haberly 1993) and similar to that observed at the Shaffer collateral/CA1 pyramidal cell synapse (reviewed in Bliss and Collingridge 2013 and in Granger and Nicoll 2014), where LTP is dependent on depolarization of the postsynaptic neurons. We thus anticipated that LTP magnitude would be correlated with initial fPSP amplitude. However, no such correlation was found (Figs. 6 and 7). We suggest that most likely explanation for this phenomenon is that the steep reduction of the fPSP amplitude observed with increasing distance between stimulating and recording electrodes reflects a reduction in the number of cells in which postsynaptic potentials are evoked, rather than the amplitude of depolarization in each cell.

### Similarity and difference between learning‐induced effects on excitatory and inhibitory synaptic transmission

As we have previously shown (Saar et al. 2012; Ghosh et al. 2015, 2016), OD learning‐induced enhancement of synaptic inhibition and excitation is widespread in the pyramidal cell population. Such a large overall increase in synaptic strength was observed following various training paradigms, in different brain structures (McKernan and Shinnick‐Gallagher 1997; Sacchetti et al. 2001, 2004; Tye et al. 2008; Yin et al. 2009). The functionality of this increase has yet to be described since its magnitude, presence in the majority of the cells, and transient nature are not consistent with the principles of classical Hebbian learning and so are novel.

We show here for the first time that: (1) Synaptic inhibition and excitation are enhanced to the same extent, and (2) while learning‐induced enhancement in synaptic inhibition can be readily explained by a process in which all inhibitory synapses are strengthened by the same additive process, enhancement of synaptic excitation is not equal for all synapses; some synapses are particularly enhanced. Taken together with the finding that only fPSPs recorded close to the stimulating electrode show enhanced excitability after learning, our results suggest that rule learning results in a net enhancement of excitability between spatially proximal pyramidal neurons, while synaptic inhibition is widespread and uniformly enhanced, and is instrumental in shaping odor representations and maintaining network stability (Poo and Isaacson 2009; Isaacson 2010). One possible explanation for the finding that rule learning is accompanied by enhanced excitatory connectivity between closely located neurons is that proximal neurons synchronize in a way that enhances the synaptic efficacy between them as opposed to innervated but distant neurons.

### Functional significance of particularly enhanced synaptic excitation in a subgroup of neurons

We previously reported that while 5 days after rule learning an increase in mEPSCs amplitude was evident in most of the recorded neurons, about 25% of the neurons showed an exceptionally great increase in the amplitude of miniature events (Saar et al. 2012). Modeling work (Reuveni et al. 2013), suggests that increase in strength such an increase of synapses in this subset of cells can serve as a long‐term whole‐cell mechanism to profoundly enhance an existing Hebbian‐type memory. This mechanism is cell‐specific rather than synapse‐specific; it modifies the channel conductance rather than the number of channels (Ghosh et al. 2015) and thus has the potential to be readily induced and uninduced by whole‐cell transduction mechanisms. Taken together with our current findings, we suggest that cells which are most likely to undergo this profound change in excitatory synaptic connectivity and subserve to maintain the memory are those with the strongest connections to proximal pyramidal neurons.

Previous studies aimed to explore which neurons in the relevant neuronal network are selected to participate in a memory trace suggest their relative CREB activity at the time of learning as a key factor (Han et al. 2007). The process is mediated by CREB's effect of enhancing neuronal excitability (Zhou et al. 2009). Importantly, a recent study strongly supports a model of fear memory formation in which intrinsic excitability determines neuronal selection, whereas learning‐related encoding is governed by synaptic plasticity (Gouty‐Colomer et al. 2016). While these studied explored the mechanism of a simple form of learning, fear conditioning, in the amygdala, a similar sequence of events may occur in the piriform cortex after olfactory discrimination rule learning. Notably, our recordings were performed 5 days after learning, when the postburst AHP is no longer reduced in neurons from trained rats (Saar et al.^1998^) and only synaptic enhancement is present. In addition, it should not be excluded that other, not localized long‐term modifications, such that cannot be identified by recording of spontaneous miniature events, are also present in the cortical network for days after learning.

To summarize, our results show that the piriform cortex network maintains its stability in the face of learning‐induced enhanced synaptic transmission by an equivalent increase in synaptic inhibition. While enhanced excitation is pronounced mainly between proximal neurons, enhanced inhibition is evenly distributed and prevents runaway synaptic overexcitation while allowing the network to store the new information related to rule learning.

## Conflict of Interest

None declared.
